# Analytical Technology in Nutrition Analysis

**DOI:** 10.3390/molecules25061362

**Published:** 2020-03-17

**Authors:** Jose M. Miranda

**Affiliations:** Laboratorio de Higiene Inspección y Control de Alimentos, Departamento de Química Analítica, Nutrición y Bromatología, Universidade de Santiago de Compostela, 27002 Lugo, Spain; josemanuel.miranda@usc.es; Tel.: +34-982-252-231 (ext. 22407); Fax: +34-982-254-592

The great challenge facing humanity in the coming decades is to secure food for the 9.8 billion people who are expected to inhabit the planet by around 2050 and 11.2 billion in 2100 [1]. To increase the food production by traditional methods to meet this demand for food is very difficult. Additionally, traditional methods of food production, both of plant and animal origin, present specific problems that make it difficult for them to meet such an ambitious increase target.

In this sense, terrestrial agriculture presents a problem because fresh water (an essential resource) is an increasingly scarce commodity, and progressive desertification of the Earth’s surface is taking place and will probably be aggravated in the future by global warming. About 45% of the world’s land surface is currently considered drylands, while 12 million hectares of land are degraded yearly through a lack of water and related processes. According to the Food and Agriculture Organization of the United Nations [2], agricultural productivity is persistently declining at over 1% per year.

With respect to food production of animal origin, this also presents specific challenges, such as the fact that intensive production methods require large amounts of land, water and feed, and some livestock (such as ruminants) produce high levels of greenhouse gas emissions. Thus, intensive methods of animal production have serious drawbacks from the point of view of environmental care. In order to properly feed such a large population, it will be necessary to increase food production while respecting ecosystems and natural resources. The current high demand for animal proteins requires that livestock is reared in large numbers over diminishing land resource which is not possible and, therefore, alternative substitutes for animal proteins needs to be embraced to overcome this problem [3].

The abovementioned fact means that demand for food produced from non-traditional sources is expected to rise in the coming decade [4]. Fortunately, nowadays increasing acceptance for novel foods is also being observed, not only in developing but also in developed countries, which is mainly influenced by consumer awareness of the nutritional benefits linked to these kinds of foods [3]. As it can be seen in Figure 1, the investigation about novel foods has experienced a dramatic increase in the last decade (about three-fold).

In addition to the need for an increase in food production, nowadays, in most countries of the world, there is a growing prevalence of chronic non-communicable diseases, many of which are diet-related [5]. As a result, there is widespread consumer demand for foods with a nutritional composition more in line with current nutritional guidelines, and which include a greater proportion of the nutrients that have a potential beneficial effect on human health, or fewer of those components that have a negative effect on human health [6]. Therefore, both because of the need to ensure food safety in food, especially in those that do not have a history of safe use. In addition, there is also a need for analytical methodologies to reliably determine both the presence of specific nutrition-related components in foods, and the effects of these dietary components on human health, in areas as diverse as lipidomics, proteomics, transcriptomics, genomics, epigenomics, or metagenomics [7,8].

To meet these needs, it is essential that we in the international scientific community work intensively to ensure safe, effective and honest food production and to protect the health of consumers. For this reason, from Molecules it was recognized the need to propose the Special Issue “Analytical Technologies in Nutrition Analysis”. This Special Issue was aimed to offer an appropriate opportunity to all the contributors to make their results and techniques more visible, and to present the most recent findings.

This Special Issue has received remarkably positive feedback, with many contributions submitted by numerous geographically diverse scientists, resulting in a collection of 10 publications, including two exhaustive review articles [9,10,11,12,13,14,15,16,17,18]. Among the contributing authors, authors can be found from Asia (China, India, and Uzbekistan), Europe (Spain), South America (Brazil and Chile) and North America (Mexico). The published articles include findings related to the comprehensive bioactive compound profile and antioxidant capacities of mamey apple (*Mammea americana*), camapu (*Physalis angulata*), and uxi (*Endopleura uchi*) that can contribute to their economic exploitation [9].

Another article described the multifunctional activity of amaranth (*Amaranthus hypochondriacus* spp.) proteins, opening the possibility that amaranth hydrolyzed with alcalase and flavourzyme to be used as a value-added ingredient with multi-functional bioactive properties [10]. Another article aimed to find an efficient extraction method and investigate some of physical and chemical parameters, like water solubility, emulsification, foaming properties, and oil-holding capacity of obtained scorpion proteins. The results obtained suggest that scorpion proteins can be considered as an important ingredient and raw material for the creation of water-soluble supramolecular complexes for drugs [11].

A fractional factorial design was used to evaluate the effects of temperature, frying time, blanching treatment and the thickness of potato slices on a very relevant potential toxic compound (acrylamide) content in crisps. The findings obtained demonstrate that acrylamide concentration remained at 70% in fried chips, and reductions took place, mainly at the intestinal phase, as a result of reaction with nucleophilic compounds [12]. *N*-carbamylglutamate, a synthetic analogue of *N*-acetylglutamate, is an activator of blood ammonia conversion and endogenous arginine synthesis. This study will provide a solid foundation for the evaluation of availability and metabolic mechanism of *N*-carbamylglutamate in animals [13].

The *Artemisia argyi* leaf has been used as a traditional medicine and food supplement in Asian countries for hundreds of years. Phytochemical studies disclosed that *Artemisia argyi* leaf contains various bioactive constituents, mainly phenolic acids, which have great potential as possible alternatives to those organic solvents in health-related areas such as food and pharmaceuticals [14]. Regarding the use of seaweeds as alternative dietary fibre sources to terrestrial vegetables, in this Special Issue an article is presented evaluating the nutritional composition and physicochemical properties of two dried commercially interesting edible red seaweeds, *Gracilaria corticata* and *G. edulis*. In view of the results, both *G. corticata* and *G. edulis* contain important nutrients for human health and are possible natural functional foods [15]. More generally, a wide review about the current knowledge surrounding the impacts of seaweeds and their derived polysaccharides on the human microbiota is also presented, in which potential benefits against chronic non-transmissible diseases were discussed [16].

Finally, two articles describing potentially beneficial food sources of fat for humans are presented. In one on them, it was concluded that refined commercial salmon oil can be transformed into a profitable source of eicosapentaenoic and docosapentaenoic acids, thus leading to a product with higher commercial value, and that this process can be optimized by using response surface methodology [17]. The last article of the Special Issue consists of a review article about avocado oil, including discussion about the extraction methods, chemical composition, and various applications of avocado oil in the food and medicine industries. Based on the available data, avocado oil has established itself as an oil that has a very good nutritional value at low and high temperatures, with multiple technological applications that can be exploited for the benefit of its producers [18].

This Special Issue is accessible thought the following link: https://www.mdpi.com/journal/molecules/special_issues/Nutrition_analysis.

As Guest Editor for this Special Issue, I would like to thank all the authors and co-authors for their contributions and all the reviewers for their effort in carefully and rapid evaluating the manuscripts. Last but not least, I would like to appreciate the hard work done by the editorial office of the Molecules journal, as well as their kind assistance in preparing this Special Issue.

## Figures and Tables

**Figure 1:**
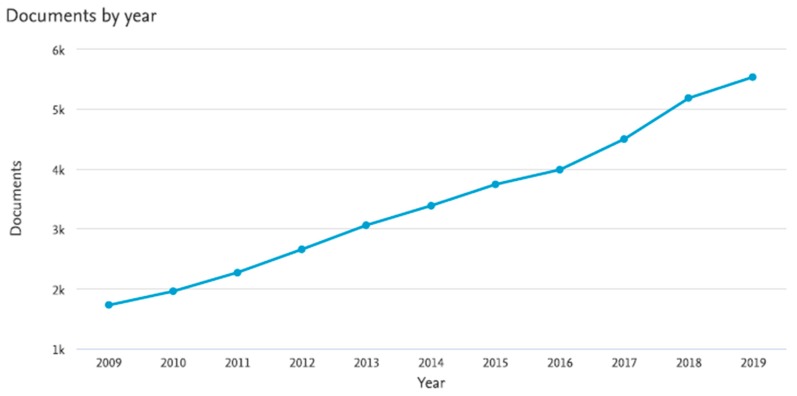
Results analysis for Scopus query “novel foods” in title, keywords or abstract section of the articles between 2009 and 2019.
